# Supplementary material for: Are carbon emissions trading and green financial instruments synergistic? -Comprehensive quantitative research based on content analysis
Source: PLoS One. 2024 Mar 7;19(3):e0298601. doi: 10.1371/journal.pone.0298601 (PMC10919601; doi:10.1371/journal.pone.0298601)
Supplement: S1 Data — (ZIP) [file pone.0298601.s001.zip › Data/地级市Z_F工作报告.zip!/╕ú╜¿/╞╬╠∩╩╨/└┤╫╘╣1⁄2╓┌║┼í╛┬φ┐╦╩2╛▌═°í┐_2018.docx]

各位代表：

　　现在，我代表莆田市人民政府，向大会报告政府工作，请予审议，并请市政协委员提出意见。

　　一、2017年工作回顾

　　过去一年，在省委、省政府和市委的坚强领导下，市政府团结依靠全市人民，深入学习宣传贯彻党的十九大精神，坚持以习近平新时代中国特色社会主义思想为指导，按照“建设机制活、产业优、百姓富、生态美的新福建”要求，围绕“坚持绿色发展，打造宜居港城、建设美丽莆田，创建美丽中国的示范区”奋斗目标，加快实施“一区、一中心、五品牌、九行动”，美丽莆田建设全面铺开，新机制新模式不断涌现，城市美誉度和影响力明显提升，经济社会发展稳中向好，较好完成了市七届人大一次会议确定的目标任务。

　　初步统计，地区生产总值2045.19亿元、增长8.4%；一般公共预算总收入205.20亿元、增长13.9%，地方一般公共预算收入136.37亿元、增长17.8%；全社会固定资产投资额2307.30亿元、增长17.1%；社会消费品零售总额695.41亿元、增长11.6%；外贸出口总额207.71亿元、增长4.3%；实际利用外资4.54亿美元、增长13.5%；居民人均可支配收入2.57万元、增长8.8%；城镇登记失业率2.5%；居民消费价格总水平上涨0.9%；年度节能减排任务基本完成。

　　一年来，我们主要做了以下工作：

　　（一）强化精准服务，经济保持平稳增长。完善产业分析推进机制，出台工业稳增长正向激励、鞋业转型升级等措施，因企施策、因企帮扶，对接省级企业技改基金12.8亿元，减轻企业负担30亿元，规模以上工业企业增加值增长8.2%。大力发展平台经济、共享经济，新增规模以上服务业企业154家，第三产业增加值增长10.8%。加快发展现代农业，成立放心食品联盟，创新天桂食品“品牌+联盟”产业融合模式，农业增加值增长3.0%。出台促进有效投资八条措施，新增“五个一批”项目1657个，25个投资超亿元制造业项目实现投产，480个重点项目和30个美丽莆田重点攻坚项目分别完成投资1046亿元、414亿元。全力防控金融风险，严厉打击恶意逃废债，不良贷款率从最高的5.65%下降到3.48%。

　　（二）实施创新驱动，产业加快优化升级。创新供应链服务平台模式，推动成立众协联、“大工美之家”，推广个性化定制，传统产业实现降本增效，规模以上工业企业利润总额增长11.4%。出台电商发展九条等措施，开展打击“仿冒鞋”“假海淘”专项行动，推动企业转型升级，让公众真心认可莆匠工艺。实施“壶兰计划”，在全省率先成立高层次人才交流协会，设立兴莆人才基金。科技创新成效明显，中科院海西研究院莆田中心竣工投用，新增一批省级智能制造试点示范企业、省科技型企业和省级工程研究中心。强化品牌创新，成功创建全国消费品工业“三品”战略示范城市、“中国油画产业之都”，莆田木雕亮相金砖厦门会晤国际舞台，莆田品牌绽放光彩。

　　（三）深化文明创建，城乡面貌明显提升。发挥敢争、敢拼、敢赢的创城精神，全民动员，全力以赴，创建全国文明城市。“11+3”特色小镇和文化名胜区创建工作全面展开，冰雪小镇方案亮相世博会，三江口镇列入全国第二批特色小镇，妈祖国际医疗健康小镇等四个小镇列入全省第二批特色小镇。实施“十个一”工程，新改建城市道路78.46公里、农村公路128公里，新建城市绿道75.9公里、口袋公园50个。中央环保反馈问题有效整改，“五小行业”整治任务全面完成，铁腕治污成效明显。全面推行河长制，创出企业河长“莆田模式”，建成市第三水厂，基本完成东圳水库水环境综合治理。生态绿心保护修复项目获评“中国人居环境范例奖”，木兰溪获评全国十大“最美家乡河”。美丽乡村建设加快推进，农村生活垃圾实现日产日清，186个村庄完成污水整治。

　　（四）突出先行先试，改革开放不断深化。创出“五融五化”就地城镇化模式，为国家新型城镇化试点提供“莆田经验”。深化“放管服”改革，在全省率先建成政务数据平台，“最多跑一趟”清单占总审批数量92.6%，“多证合一”改革走在全国前列，新增市场主体5.4万户。国企改革取得突破，运作模式更加灵活，市属国有企业总资产增长25%。基本完成农村土地承包经营权确权登记。在全省率先试点建设社区综合受理平台和网格化社会服务管理信息平台，严重精神障碍患者网格化“一历五单”救治机制在全国推广，食品药品网格化监管“三个三”模式在全省推广。莆田港口岸扩大对外开放获国家批复。成功举办第二届世界妈祖文化论坛，开展妈祖千年首巡东南亚、二十年赴台再巡安等活动，妈祖文化再耀“海丝”。承办第三届中欧城市可持续发展论坛，入选中国国际城市发展联盟，与澳大利亚朗塞斯顿市建立友好城市关系。

　　（五）坚持共享发展，人民生活更加殷实。民生支出173亿元、增长10.7%。办好19项为民办实事。新增城镇就业人数1.89万人，转移农村劳动力2.06万人，造福搬迁3051人，现行标准下的贫困人口、贫困村全部实现脱贫。完成全民参保登记，城乡居民养老保险参保缴费率居全省前列。教育强市迈出坚实步伐，新建幼儿园项目17个、中小学项目93个，新增中小学学位8500个，启动东南医学院筹建工作，莆田学院、湄洲湾职业技术学院迁建加快推进。在全省率先成立药联体，实现药品阳光采购、医保管办分离和业务同城通办；建成首批11个市级临床重点专科，改造提升100家村卫生所，千人均病床位达5.16张。实施莆仙戏振兴计划，开展“美丽莆田”系列文艺汇演，建成投用市博物馆、图书馆、非遗展示馆。全民健身活动广泛开展，成功举办金砖国家少年足球邀请赛、“妈祖杯”海上丝绸之路国际羽毛球挑战赛、市第四届运动会等重要赛事。完成第三次全国农业普查。“七五”普法顺利开展。成功防御“纳沙”等台风。深化平安莆田建设，深入开展安全生产大检查，信访、综治、维稳工作扎实推进。荣获新一届省级双拥模范城。民族宗教、粮食安全、统计、档案、工会、妇女儿童、青少年、残疾人、红十字会等工作取得新成效。

　　（六）从严从实治政，政府建设全面加强。扎实推进“两学一做”学习教育常态化制度化，深化“五抓五看”要求，推动“两个责任”落细落实。全面加快法治政府建设，加强政务公开、信息公开，认真执行人大决议，主动向政协通报情况，自觉接受社会监督，深化与法院、检察院联系互动，办理市人大建议212件、市政协提案212件，办结率均为100%。在全省率先出台机关效能建设正向激励和容错纠错举措，形成一批典型工作法，“马上就办、真抓实干”的氛围更加浓厚。严控“三公”经费支出，完成党政机关公务用车制度改革。强化行政监察和审计监督，廉政建设和反腐败工作扎实推进。

　　各位代表，过去一年是新一届政府的开局之年，一年来，我们举全市之力，勇于挑战不可能，一举创成全国文明城市，世界妈祖文化论坛永久会址建设创出“湄洲速度”，获得第五届世界佛教论坛举办权，各项事业发展不断取得新突破，全市上下风清气正、心齐气顺，干事创业热情空前高涨。成绩的取得，是我们坚持以习近平新时代中国特色社会主义思想为指导，认真落实习近平总书记对福建工作重要指示精神的结果；是省委、省政府关心支持和市委坚强领导的结果；是全市上下团结拼搏、社会各界人士和海内外朋友鼎力支持的结果。在此，我代表市人民政府，向全市人民，向各民主党派、各人民团体和社会各界，向驻莆部队、武警官兵，向港澳台同胞、海外乡亲和国际友人，表示衷心的感谢！

　　同时，我们也清醒地认识到存在的不足和问题。主要是：发展质量和效益还不高，实体经济水平有待提升；城市聚集力吸引力还不够强；民生领域存在不少短板，群众在教育、医疗、养老等方面面临不少难题；生态环境保护压力较大；一些部门还缺乏“逢山开路、遇水架桥”的精气神，营商环境仍需优化；对照市七届人大一次会议确定的目标任务，还有3.1%的子项目未完成。我们必须直面问题，创新举措，切实加以解决。

　　二、2018年主要工作

　　2018年，是全面贯彻党的十九大精神的开局之年，是改革开放40周年，是决胜全面建成小康社会、实施“十三五”规划承上启下的关键一年，也是我省实现赶超目标的关键一年。政府工作的总体要求是：全面贯彻党的十九大精神，以习近平新时代中国特色社会主义思想为指导，坚持稳中求进工作总基调，坚持新发展理念，紧扣社会主要矛盾变化，按照高质量发展的要求，统筹推进“五位一体”总体布局和协调推进“四个全面”战略布局，认真落实中央经济工作会议、省委十届五次全会和市委七届五次全会精神，紧紧围绕“再上新台阶、建设新福建”和创建美丽中国示范区的目标，坚持以供给侧结构性改革为主线，把高质量发展与实现赶超有机统一起来，着力强产业提后劲，着力增创新优供给，着力促改革添动力，着力抓城乡促提升，着力补短板惠民生，着力美生态固优势，着力防风险守底线，进一步强化压实责任，强化正向激励，强化工作抓手，推动质量变革、效率变革、动力变革，促进经济社会持续健康发展，推动美丽莆田建设迈出新步伐。主要预期目标是：地区生产总值增长10.5%左右；一般公共预算总收入增长11%，地方一般公共预算收入增长11%；固定资产投资增长18%；社会消费品零售总额增长12%；外贸出口总额增长5%；实际利用外资增长10%；居民消费价格涨幅控制在3%左右；城镇登记失业率控制在3%以内；居民人均可支配收入增长11%；完成节能减排降碳任务。

　　实现上述目标，我们要在市委的坚强领导下，以赶超的气势、创新的理念、系统的思维、担当的魄力，重点做好以下工作：

　　（一）深化供给侧结构性改革，把着力点放在实体经济上，建设协同发展的产业体系，推动增量崛起存量变革

　　加快培育发展新兴产业。围绕打造电子信息、化工（纺织化纤）新材料千亿产业集群和医疗健康、高端装备、海洋五百亿产业集群，瞄准国内空白、全国领先，依托产业龙头，实施精准策划招商，集聚要素资源，制定路线图，推动倍增发展。电子信息产业，做强华佳彩面板、砷化镓、HDT高效太阳能电池、元生智汇龙头，建设佳华超金属膜基材、宽幅偏光片、显示模组、中电互联网信息产业园、创世纪超算中心等项目，铸全新型显示、集成电路、太阳能电池和智能终端链条，打造新一代电子信息产业基地、智能终端产业基地和互联网信息产业中心。产值增长30%以上。化工（纺织化纤）新材料产业，以永荣CPL为龙头，策划引进下游纺丝、织造等项目，建设国际尼龙城；以华峰集团、赛得利、华源工贸为龙头，发展织造、染整、高端面料、循环利用、智能装备全产业链；以三棵树为龙头，集聚相关企业，延伸新型建材、健康大家居等产业链，打造国际涂料城。产值增长20%以上。医疗健康产业，以妈祖国际健康城为龙头，联手国药、新希望、树兰、威高、英国爱丁堡大学等国内外龙头以及台湾海峡两岸医事交流协会，建立百亿产业基金，加快建设高端专科医院集群、先进药械制造基地、康养中心、医学交流转化中心、医疗大数据中心、医生集团总部，打造中国第一康城和两岸健康产业先行区。产值增长30%以上。高端装备产业，依托云度等龙头，借智中国电动汽车百人会，吸引一批行业领军企业入驻，做强“三电系统”产业链，建设新能源汽车产业园，打造“电动福建”示范基地；依托上海电气、海山机械等龙头，延伸港口装备、环卫设备产业链，建设综合型海上风电设备产业基地、先进环卫设备产业基地。产值增长30%以上。

　　推进传统产业内涵发展。围绕打造鞋服、工艺美术、食品加工、建筑等千亿产业集群，鼓励企业联盟创新、联合创牌，打好“互联网+”“标准化+”“数字化+”组合拳，对标提质，推动存量变革。鞋服产业，依托鞋业供应链服务平台，聚合鞋业采购需求和上游供应资源，嫁接先进技术、高端材料、智能制造，打造个性化定制和“中国军鞋莆田造”区域品牌，让世界爱上莆田好鞋。产值增长15%以上。工艺美术产业，依托“大工美之家”“仙作供应链”“金银谷互联网+”等公共平台，嫁接高端资源，建设国家级质量检测鉴定中心、研发设计院，构建集研发、设计、生产制造、评价鉴定、监管服务、会展节庆于一体的现代产业体系，打响莆田好家具、好珠宝区域品牌。产值增长25%以上。食品加工产业，以雪津为龙头，发展麦芽、麦汁、啤酒、包装、体验旅游全产业链，做强雪津食品产业园；以和润粮油、卯兴麦芽为龙头，加快东吴食品工业园建设；立足绿色食品品牌，整合资源，抱团协作，发展精深加工。产值增长15%以上。建筑产业，支持发展总部经济，培育引进一批现代化建筑企业，推广装配式建筑建造方式，推动工程总承包试点企业扩大规模、资质升级、做优做强。产值增长15%以上。木材产业，围绕打造全国一流的进口集散、仓储物流、研发设计、加工制造、会展商贸全产业链的海丝木业城，建设无醛木业产业园，同时争取宜华木业和产业链龙头入驻回归，加快秀屿木材贸易加工区转型升级，构建新模式，抢占新高地。产值增长30%以上。

　　加快发展现代服务业。推动生产性服务业和生活性服务业并进，大力发展数字经济、平台经济、共享经济、智能经济、总部经济、楼宇经济、会展经济，着力拓展数字消费、电子商务、现代物流、文化创意等新兴服务业。发展服务型制造，引导制造业工贸分离，大力引进总部型企业。加快国家电子商务示范城市建设，扶持壮大电子商务平台。成立“智慧油站”联盟，搭建集“供油、结算、监管、金融、共享”的生态服务平台，促进民营加油站资源共享和行业整合提升。推进区域金融创新，大力发展普惠金融、绿色金融、科技金融，引进境内外金融机构来莆设点展业，鼓励企业挂牌上市，做大金融总量；持续防范和化解金融风险，坚决守住不发生系统性金融风险底线。加快实施全域旅游行动计划，建设旅游提升“五大工程”，促进跨界融合，培育一批有特色、有影响、有效益的精品，旅游总收入增长20%以上。

　　全方位营造创新创业生态。发挥莆田高新区等专业园区产业“孵化”作用，建设中科院STS莆田分中心，筹建新工科学院、中电智能制造研究院、新型显示研究中心，培育提升智能制造、集成电路等一批共性技术研发与转化平台。实施高成长企业和高新技术企业“双高”培育工程，启动“瞪羚”计划，建立“独角兽”种子企业储备库，推动潜在企业和种子企业快速成长。扎实推进国家知识产权试点城市建设，创新成果转化股权和分红方式，完善知识产权创造、保护和运用体系。实施人才集聚提升工程，建设高端人才社区，打造更多人才社群，优化“引育留用”全链条人才生态，让人才在莆田舒心工作、大显身手。

　　（二）开展城乡规划建设管理提升年行动，推动乡村振兴，建设更加宜居宜业的美丽莆田

　　提升规划设计水平。抢占城市群发展先机，以系统性、整体性、生长性的城市发展理念，修编城市总体规划，完成“莆田2049”发展战略研究、综合交通规划、绿心规划、“城市双修”系列规划，以及历史文化名城名镇名村、传统村落保护规划。全面开展重点地区、重点地段城市设计，努力提高建筑设计水平。建设“多规合一”管理平台，创新规划管理和服务机制。

　　提升城市承载力。打造内外通畅的交通格局，加快福厦客专建设和妈祖机场、铁路货运专线前期工作，加快联十一线、纵一线等城市对外交通干道，以及木兰大道、八二一大街南伸等城市主干路网建设，改造提升荔园路、城港大道、黄石高速路口、动车站站前路网等项目，打通学园街北伸等城市“断头路”，改善道路交通微循环。完善城市配套设施，全面开展地下管网普查，建立管网信息平台，加快实施污水处理三年行动计划，完成主城区污水收集和雨污分流改造，新建污水管道90公里，提标改造污水处理厂4座，建设城市会展中心、植物园、餐厨垃圾处置场、危险废物处理厂。推进城市东进南拓，启动绿心保护核心区建设，开发大学城片区、木兰溪沿岸地区、高铁站周边片区、莆一中新校区周边地区等城市重要功能片区，全力支持仙游加快建设。

　　提升城市宜居度。推进城市修补，创建绿色社区、绿色机关、绿色校园，新建改造城市公园10个，口袋公园50个，新增生态绿道30公里，建设“一区一溪两路两桥三场”夜景工程，美化城市门户地区、主要街道的市容市貌，刷新城市颜值。完善城市公共交通，优化公交线路，实现工业园区公交站线全覆盖，发展城市公共自行车系统，建设一批人行过街设施，创建人行道整治“示范街”，加快城市轨道交通前期工作，让群众出行有更多的选择。实施城市环境综合整治行动计划，改造一批城市棚户区、老旧小区，开展城市生活垃圾分类试点，全面推进厕所革命。开展古城、古镇、古村保护修缮行动，保育城市文脉。

　　提升城市管理水平。推进精细化管理，深化城市管理执法体制改革，开展城市管理国际化对标行动，全面整治“两违”、物业管理等薄弱环节，下足“绣花”功夫，全力破解一批突出难题。加快智慧城市建设，探索建立“互联网+全民城市治理”模式，建设智慧型城区，拓宽市民参与渠道，让人人都是城市管理者、人人都是智慧服务受益者。

　　全面加快特色小镇建设。坚持“产业为根、文化为魂、生态为基、人才为本”的理念，加快高端要素集聚，带动公共设施和服务向农村延伸，推动城乡融合发展。冰雪小镇，围绕打造国内最具特色的LNG冷能资源综合利用基地、最具吸引力的亚热带冰雪体育休闲旅游目的地，开工建设换冷站、大道滑冰馆、滑雪馆等项目。金银谷，围绕建设“全球最高端、业态最完整、配套最齐全”的金银珠宝第一镇，搭建联合采购平台，建立产业发展引导基金，吸引知名品牌加盟、高端制造进驻，开工建设金银珠宝展示中心，举办海上丝绸之路国际珠宝节，推动品牌集聚、跨界融合。广化寺佛教名胜区，完善海丝沿线国家佛教文化中心、海峡两岸佛教交流中心的策划定位，以举办第五届世界佛教论坛为契机，建设提升南湖公园、广化路、停车场以及绿化景观等项目，推动周边片区有机更新，带动城市功能转型升级。仙作、艺雕、啤酒、鞋艺等特色小镇和文化名胜区，进一步提升站位，加快规划建设，培育产业生态，形成集聚带动效应。

　　大力实施乡村振兴战略。坚持质量兴农，巩固粮食安全，扶持发展10个现代农业产业园，发展设施农业、农产品加工业、休闲农业，促进农村一二三产业融合发展，构建现代农业产业体系。坚持绿色兴农，加快美丽乡村、幸福家园建设，打造北部山区富美乡村示范带等一批生态风景线，发展乡村旅游。建立“路长制”，推进“四好农村路”建设。全面开展农村人居环境整治行动，全市75%以上乡镇建成污水处理设施，260个行政村完成生活垃圾治理提升。完成农村集体资产清产核资、土地承包经营权确权颁证，推进农垦市场化、社会化改革。培养造就一支懂农业、爱农村、爱农民的“三农”工作队伍，创新乡村治理体系，不断提高乡村社会文明程度。

　　（三）培育和践行社会主义核心价值观，大力弘扬妈祖文化，提升文化产业，不断夯实文化强市根基

　　全力保护好湄洲岛。牢记习近平总书记嘱托，全面落实《关于切实保护好湄洲岛的若干意见》，创建国家级妈祖文化生态保护实验区。强化岛内、近岸海域和国家级海洋公园的生态保护，依法严禁采砂挖泥行为，强化“两违”管控，守住生态红线、天际线、海岸线。完善慢行系统，推广新能源汽车，打造无人驾驶示范岛。推动世界妈祖文化论坛国际化、品牌化，提升妈祖文化对台和对外的影响力、感召力，让妈祖精神扬帆“海丝”。建设朝圣旅游码头，保护妈祖历史古迹，植入旅游新业态，打造多层次旅游产品体系，创成国家5A级旅游景区。

　　扩大公共文化供给。深入开展群众性精神文明创建活动，切实将社会主义核心价值观转化为市民的情感认同和行为习惯。大力实施文化惠民工程，打造“百姓舞台”等一批公共文化服务示范项目。建成投用市科技馆，推进市艺术学校新校、市档案馆迁建工程，启动市美术馆建设。坚持以人民为中心的创作导向，推出一批走心的本土优秀文化精品力作。弘扬优秀传统文化，加强文化遗产传承保护，启动申报国家历史文化名城。

　　培育新型文化业态。持续发挥“文化+”功能，推进文化产业与创意设计、影视演艺、休闲旅游、节庆会展、数字动漫等产业的联动发展。深入实施文化产业龙头促进计划，鼓励组建综合性企业集团，打造一批行业标杆，提升一批文化产业园区，力争文化产业增加值占地区生产总值的比重超过10%。

　　（四）深化简政放权、放管结合、优化服务改革，全力打造审批更少、流程更优、体制更顺、机制更活、效率更高、服务更好的营商环境

　　深化“放管服”改革。巩固“多证合一”改革成果，推行“证照分离”改革，全面落实市场准入负面清单制度，最大程度释放全社会投资潜力。实施“一号一窗一网”，推行“双随机、一公开”监管机制，全面提升行政服务效能。实施大数据战略，加快建设数字莆田，推进全市公共信息资源汇聚和互联互通，打造公共智能服务模式，推进商事登记全程电子化，逐步实现企业事务全网通办，电子证照应用率达80%以上，“一趟不用跑”和“最多跑一趟”事项超过95%。

　　做强做优做大国有企业。推动市属国有企业建立现代企业制度，完善法人治理结构，创新人才引进、正向激励机制，加快实体化市场化运作，提高运营水平和市场拓展能力，在全市赶超发展中挑大梁、当先锋，力争国有企业总资产突破千亿元，实现莆田国企跨越发展。

　　营造公平竞争的市场环境。完善信用信息共享平台，建立社会组织统一社会信用代码数据库，打造全程质量可追溯体系，创建全国信用体系建设示范城市，让诚信成为城市品牌和全民自觉。全面清理涉企收费、摊派事项和各类达标评比活动。积极借鉴自贸区建设经验，健全与国际接轨的营商机制，创造无障碍的投资环境，吸引海内外“投资客”。

　　激发和保护企业家精神。加大鼓励创新创业的政策支持和载体建设，实施企业家培训计划，加快培育新生代，提升企业家的现代理念和创新能力。营造鼓励创新、宽容失败的文化和社会氛围，靠前服务，诚心帮助，多一些成全，少一些求全，真诚坦荡同企业家交朋友，加快构建亲清新型政商关系，弘扬天下莆商的优秀特质，激发企业家爱拼敢赢、追求卓越的活力。

　　（五）主动融入“一带一路”倡议，发展更高层次的开放型经济，推动形成全面开放新格局

　　积极参与“一带一路”建设。加强与海丝沿线国家和地区的交流合作，鼓励优势企业通过并购、技术合作等方式走出去，在境外建设国际营销网络体系。加快建设跨境电商产业园，重点发展市场采购贸易、外贸综合服务等新业态，扩大“生意圈”。以妈祖文化为纽带，增进经贸合作，发展旅游贸易。推进侨务引智工作，培养侨界新生力量。

　　提高港口对外开放水平。推动罗屿9号泊位列入国家40万吨铁矿石码头布局规划，发挥港口深水中转优势，发展水水中转、海铁联运，加强与江西、湖南、台湾、海丝沿线港口城市的合作交流，推动建设“陆地港”“飞地港”，大力发展矿石集散、煤炭混配、保税仓储、货代船代和期货金融服务等，积极培育港口要素新业态。对标国际先进规则，建设海关特殊监管场所，推进港口贸易投资自由化和便利化，提升港口综合竞争力。

　　深入开展莆台交流合作。发挥台资龙头企业作用，建设莆台电子信息、健康产业、电商物流、农业合作基地，培育两岸邮轮旅游，促进莆台产业深度融合。推动莆台朝圣客运直航常态化。建设对台交流基地和台湾青年体验交流中心，拓展民间基层交流交往，深化“莆台缘”。加强莆港澳经贸、人文和产能合作。

　　（六）牢固树立和践行绿水青山就是金山银山的理念，加大生态环境保护治理力度，为市民提供更多优质生态产品

　　全力打好污染防治攻坚战。扎实抓好中央、省环保督察反馈问题整改工作，巩固“五小行业”专项治理成果。深化工业废气、城市扬尘、机动车污染防治，专项治理化工等行业挥发性有机物。全面落实河长制，加强木兰溪、萩芦溪等重点流域水污染综合整治，提升南北洋河网等6条小流域水质，基本消除城市建成区内黑臭水体，全面消除劣Ⅴ类小流域，全市工业园区企业污水全部接入管网处理。建设土壤环境监管网络，深入推进土壤污染防治。

　　强化重要生态系统保护和修复。完成生态保护红线调整划定，建立河岸生态保护、饮用水源地保护、地下水警戒保护三条蓝线管理制度，建设安全生态水系50公里，创成全国水生态文明试点城市。加快东圳水利枢纽引水配套工程，推进双溪口至东圳水库引水工程前期工作。加强森林生态治理，实施造林绿化3.8万亩以上、重点生态区位商品林赎买3000亩以上，创建国家森林城市。加强矿山生态治理。严格落实海洋功能区划，开展“海漂垃圾”、水产养殖整治，保护和修复湿地，让莆田碧波荡漾、海天一色。

　　改革生态环境监管机制。开展自然资源资产统一确权登记，编制自然资源资产负债表。建立环境行政执法与司法高效联动机制，复制推广生态环境损害赔偿试点经验。建立资源环境承载能力监测预警协调机制以及排污权抵押贷款、环境治理基金等绿色金融机制，激发企业治理污染积极性。

　　（七）在发展中保障和改善民生，加快补齐民生社会事业短板，使人民群众获得感、幸福感、安全感更加充实、更有保障、更可持续

　　打好精准脱贫攻坚战。坚持把提高脱贫质量放在首位，注重扶贫同扶志、扶智相结合，以更加集中的支持、更加有力的举措、更加精细的工作，突出抓好返贫人口动态监测和管理，全面落实产业、就业、教育、金融等扶贫措施，建立健全光伏扶贫、乡村旅游等资产性收益稳定脱贫机制，激发贫困人口内生动力。健全基本医疗保险、大病保险、医疗救助、医疗叠加保险、医疗综合补助五重保障制度，构建更加完善的脱贫保障体系，让群众脱得了贫、致得了富。

　　加快教育强市建设。着力解决中小学生课外负担重、“择校热”“大班额”等突出问题，解决好婴幼儿照护和儿童早期教育服务问题。推进学前教育普惠健康发展，新建一批普惠性幼儿园，构建公办和民办并举的学前教育服务体系。实施城镇中小学扩容工程、初中“壮腰”工程、优质高中建设工程，开工建设莆一中新校区。加快推进“两院”新校区建设和东南医学院筹建，支持莆田学院转型发展，提升湄职院等职业教育内涵，深化产教融合。推动教师“县管校聘”改革，构建教师编制能进能出、职称能上能下、综合奖补能多能少的用人机制。全面实施“三名”工程，继续加大高层次教师人才引进力度，推进“名校托管弱校”模式，全面提升优质教育覆盖面，让更多孩子在家门口就可以上好学。

　　实施健康莆田战略。启动市老年、妇幼保健等一批专科医院建设，新增病床位850张。实施医药卫生人才“三大工程”和基层医疗卫生服务能力提升工程，标准化改造100家村卫生所。启动中医固本三年行动，新增中医馆20家。深化医药卫生体制改革，开展县域医共体建设试点，组建特色专科联盟，建设和发展多种形式医联体，推行乡村卫生服务一体化和家庭医生签约服务，让健康惠及全民。深入创建国家食品安全示范城市。启动国家卫生城市创建工作。推动全民健康与全民健身深度融合，建好群众身边体育设施，举办国际性赛事，发展体育产业。

　　构建多层次社会保障体系。加快建设就业信息化平台和镇街就业服务所，注重解决结构性就业矛盾，提供全方位公共就业服务，新增城镇就业1.7万人。进一步实施全民参保计划，推动进城务工人员参加企业职工养老保险，实现基本养老保险参保人数超200万人。构建多层次养老服务体系，优先发展居家社区养老服务，推广购买服务、公建民营、民办公助等方式，培育专业化服务组织，大力推进医养结合试点工作，让每一位老年人都能老有所养、老有所依、老有所乐。完善社会救助、社会福利、慈善事业、优抚安置等制度，健全农村留守儿童和妇女、老年人关爱服务体系。建立多主体供应、多渠道保障、租购并举的住房制度，完善促进房地产市场平稳健康发展的长效机制，发展住房租赁市场，实施安居工程，让广大群众，特别是困难群众住有所居、住得温暖。

　　打造共建共治共享的社会治理格局。牢固树立安全发展理念，建立突发事件应急处理机制，坚决遏制重特大安全生产事故，提升防灾减灾救灾能力。推进社会治理专项行动，提升社会治理社会化、法治化、智能化、专业化水平。深入推进“七五”普法，提高全民法治意识，培育自尊自信、理性平和、积极向上的社会心态。推进移风易俗，引导社风民风向上向善。做好村级委员会换届选举工作。创新群众工作机制，充分发挥工会、共青团、妇联等群团组织联系群众的桥梁纽带作用。持续推动“阳光信访”“法治信访”建设，完善社区治理体系，实现政府治理、社会调节、居民自治良性互动。推进军民融合深度发展，争创全国双拥模范城“六连冠”。

　　三、建设人民满意

　　的服务型政府

　　各位代表！新时代要有新作为，我们必须时刻牢记职责使命，时刻秉持严实作风，时刻坚守为民情怀，不断提升政府治理能力现代化水平，努力建设人民满意政府。

　　我们一定旗帜鲜明，立场坚定，切实做到政治过硬。把政治建设摆在首位，深入学习宣传贯彻党的十九大精神，坚决维护习近平总书记在党中央和全党的核心地位，坚决维护以习近平同志为核心的党中央权威和集中统一领导，牢固树立“四个意识”，坚定“四个自信”，坚决贯彻执行党中央国务院、省委省政府和市委决策部署。深入推进“两学一做”学习教育常态化制度化，扎实开展“不忘初心、牢记使命”主题教育，严格落实意识形态工作责任制，全面加强党组建设，筑牢战斗堡垒。

　　我们一定增强本领，改进作风，切实做到勤政为民。适应新时代提出的新要求，既要政治过硬，又要本领高强，善于学习，勇于实践，培养专业能力，弘扬专业精神，补齐能力短板。坚持敢于创新、勇于担当，增强狠抓落实本领，善于摸实情、出实招、重实效，当好“操盘手”和“施工员”，扑下身子、苦干实干，以钉钉子精神，做实做细做好各项工作，真正把群众的期望变成生活的现实。坚持从群众最关心、最操心、最烦心的事情做起，把更多财政支出优先投向民生事业，紧盯30项为民办实事项目，一件一件抓落实，一事一事见成效，让群众真切感受到政府的用心和善政。

　　我们一定公正用权，接受监督，切实做到依法行政。坚持科学决策、民主决策，深化政务公开和政府信息公开，增加透明度，保障人民群众的知情权、参与权、表达权、监督权，让人民群众知道政府在想什么、做什么，赢得人民群众的充分理解、广泛支持和积极参与。依法接受市人大监督和市政协民主监督，重视司法、舆论、社会监督，加强行政监察和审计监督，习惯在受监督和约束的环境中工作生活。全面建立政府法律顾问制度。强化行政决策合法性审查、行政执法案件评查，规范执法行为。建设诚信政府，信守政府承诺，以政府诚信带动社会诚信。

　　我们一定正风肃纪，弘扬正气，切实做到清正廉洁。严格落实党风廉政建设工作责任制，以坚如磐石的决心，坚定不移反腐倡廉，深化标本兼治，巩固拓展落实中央八项规定精神成果，驰而不息纠正“四风”，全力整治群众身边腐败问题，加强对重点领域、重大项目、关键环节、重要岗位权力运行的监督制约，推动形成清清爽爽的同志关系。关心关爱基层干部，全力推行正向激励和容错纠错机制，态度鲜明地为那些苦干实干、不谋私利的干部撑腰鼓劲，最大限度调动广大干部的积极性、主动性、创造性，营造致力创新破难、奋力跨越赶超的浓厚氛围，让创新者脱颖而出，让实干者人生出彩。

　　各位代表！幸福都是奋斗出来的。让我们更加紧密地团结在以习近平同志为核心的党中央周围，在市委的坚强领导下，一步一个脚印，踏踏实实干好每一项工作，奋力谱写莆田发展新篇章，为创建美丽中国的示范区而不懈奋斗！
